# Application of Behavioral Science in Digital Therapeutics for Individuals With Prediabetes: Scoping Review

**DOI:** 10.2196/78891

**Published:** 2025-09-29

**Authors:** Zhuyanyang Pan, Xueli Li, Qiaoyuan Yan, Na Zeng

**Affiliations:** 1 College of Medicine and Health Sciences China Three Gorges University Yichang China; 2 The First College of Clinical Medical Science China Three Gorges University, Yichang Central People's Hospital Yichang China

**Keywords:** behavioral science, behavior change techniques, prediabetes, digital therapeutics, mobile health, scoping review, mobile phone

## Abstract

**Background:**

Digital therapeutics are increasingly used to manage prediabetes due to their accessibility and potential for personalization. Their success depends heavily on applying behavioral science and integrating theoretical models into digital platforms. However, there has not been a comprehensive account of how behavioral science has been used in digital therapeutics for individuals with prediabetes.

**Objective:**

This scoping review aimed to examine the use of behavioral theories and techniques in digital therapeutic interventions for individuals with prediabetes, and to identify opportunities to optimize theory-driven and technology-supported strategies.

**Methods:**

A scoping review was conducted following the Arksey and O’Malley framework and guided by the PRISMA-ScR (Preferred Reporting Items for Systematic Reviews and Meta-Analyses extension for Scoping Reviews) checklist. We systematically searched PubMed, Embase, Web of Science, the Cochrane Library, Scopus, CNKI, and VIP Database for Chinese Technical Periodicals for studies published up to March 10, 2025. Eligible studies included adults (≥18 years) with prediabetes, as defined by the American Diabetes Association, and examined digital therapeutic interventions informed by behavioral science. All study designs were eligible; included studies were screened, and key characteristics were charted.

**Results:**

Of the 21 included studies, 17 were randomized controlled trials. The most frequently used behavioral theories were social cognitive theory, theory of planned behavior, and transtheoretical model; however, 11 studies applied behavior change techniques without explicitly stating a theoretical framework. In terms of delivery, digital modalities often comprised smartphone apps (14/21, 67%), human coaching (13/21, 62%), messaging tools (9/21, 43%), wearable devices (9/21, 43%), and web platforms (3/21, 14%). About behavior change techniques, the most frequently used were self-monitoring of behavior (19/21), instruction on performing the behavior (16/21), goal setting (15/21), information about health consequences (15/21), and unspecified social support (11/21). Across studies, outcomes were typically assessed for metabolic and body composition (19/21), glycemic control metrics (17/21), cardiovascular risk and physiological function metrics (16/21), behavioral and cognitive intervention indicators (11/21), and, less frequently, comprehensive health outcome measures (2/21).

**Conclusions:**

Behavioral science plays a crucial role in developing effective digital therapeutics for individuals with prediabetes. However, greater clarity in theory selection, better integration between models and digital functions, and more culturally inclusive research are needed to improve the scalability and impact of these interventions.

## Introduction

Prediabetes is a metabolic abnormality in which blood glucose levels are elevated above normal but do not meet the diagnostic threshold for diabetes [1]. According to American Diabetes Association diagnostic criteria [1], prediabetes is defined by impaired fasting glucose (fasting plasma glucose 100-125 mg/dL [5.6-6.9 mmol/L]); impaired glucose tolerance (2-hour plasma glucose of 140-199 mg/dL [7.8-11.0 mmol/L] during a 75-g oral glucose tolerance test); or these 2 abnormalities may also co-occur; additionally, glycated hemoglobin (HbA_1c_; 5.7%-6.4%) is regarded as prediabetes as well. Prediabetes is rising rapidly alongside the global epidemic of metabolic diseases [2]. The IDF (International Diabetes Federation) Diabetes Atlas (11th edition) estimates that 635 million adults have impaired glucose tolerance and 488 million have impaired fasting glucose; these figures are projected to rise to 847 million and 648 million by 2050 [3]. As an inevitable stage in the development of diabetes, prediabetes is associated with an annual progression rate of 5%-10%, with approximately 70% eventually developing diabetes [4], along with heightened risks of chronic conditions such as cardiovascular disease and metabolic syndrome [5]. These trends significantly contribute to the global economic burden of chronic diseases; in 2024, international health expenditure on diabetes was approximately US $1.01 trillion [3].

Although existing studies have confirmed that lifestyle interventions grounded in behavioral strategies can effectively delay or even reverse disease progression [2,6], traditional intervention models face limitations in terms of reach, efficiency, and sustainability. There is an urgent need to explore more effective prevention and control strategies. As a product of the digital era, digital therapeutics offer unique advantages [7]. By integrating modern information technologies, such as mobile internet, big data, and artificial intelligence, and leveraging platforms such as mobile apps, wearable devices, and remote monitoring systems, digital therapeutics enable real-time monitoring and intervention in individual health behaviors [8]. For individuals with prediabetes, goal-setting techniques can facilitate personalized dietary management [9]. Self-monitoring technologies can support exercise tracking systems [10]. Furthermore, reminder and incentive strategies can deliver stepped educational interventions [11]. These functional modules translate behavior change techniques (BCTs) into actionable, measurable units, enabling proactive intervention through continuous monitoring and instant feedback, thereby significantly expanding the scope and effectiveness of health management strategies [7].

However, from a theoretical standpoint, the effectiveness of digital therapeutics is fundamentally rooted in behavioral science. Behavioral science, an interdisciplinary field that encompasses psychology, sociology, and economics, investigates the patterns and determinants of human behavior. It provides a theoretical framework for designing interventions by elucidating the cognitive, emotional, and environmental mechanisms underlying individual and group behaviors [12]. In the field of public health, classic theories such as social cognitive theory (SCT) [13], the theory of planned behavior [14], the health belief model [15], prospect theory [16], and nudge theory [17] have been shown to effectively facilitate the adoption of healthy behaviors through BCTs. Intervention designs grounded in behavioral science have been shown to significantly improve self-management behaviors in individuals with prediabetes. The US Diabetes Prevention Program, based on behavioral science theories and BCTs, successfully enabled behavior change in diet and physical activity among individuals with prediabetes. This program resulted in a 58% reduction in diabetes incidence, demonstrating the effectiveness of behavioral science in disease management [18].

Nevertheless, a lack of systematic synthesis remains regarding how behavioral science is applied within digital therapeutics. It is also unclear whether such applications can effectively drive behavioral change and improve clinical outcomes. In light of this gap, this study adopts a scoping review approach. It will examine the theoretical foundations, technological implementation forms, core intervention components, and clinical evidence of digital therapeutics targeting individuals with prediabetes. This review aims to clarify the current state of progress and limitations in interdisciplinary research and to provide guidance for the development of theoretically coherent, technologically adaptable, and scalable precision intervention strategies.

## Methods

### Study Design

This study was conducted following the framework proposed by Arksey and O’Malley [19] for scoping studies and reported by the PRISMA-ScR (Preferred Reporting Items for Systematic Reviews and Meta-Analyses extension for Scoping Reviews) guidelines [20]. Details of the identification, screening, and study selection process are provided in Multimedia Appendix 1— the PRISMA-ScR checklist.

### Search Strategy

The authors’ predetermined eligibility criteria for paper selection. A reproducible search strategy was used to identify evidence on applying behavioral science in digital therapeutics for individuals with prediabetes. We selected a range of representative databases with broad coverage for literature retrieval, including PubMed, Embase, Web of Science, the Cochrane Library, Scopus, and Chinese databases such as CNKI and VIP Database for Chinese Technical Periodicals. The search covered papers published from the inception of each database up to March 10, 2025.

In PubMed, the search strategy combined Medical Subject Headings and free-text terms related to behavioral science, digital therapeutics, and prediabetes. Search terms were developed iteratively. We began with seed papers, expanded our research by tracking reference lists, and identified keywords and Medical Subject Headings or EmTree terms. Pilot searches refined Boolean and proximity operators across databases. The review team finalized the search strings. Reference lists of relevant publications were manually screened to identify additional studies. The detailed search strategy for PubMed is presented in Textbox 1, and the complete list of search terms for other databases is provided in Multimedia Appendix 2—search strategies for all databases.

All citations retrieved from each database were imported into EndNote (version 21; Clarivate). Duplicates were identified using EndNote’s built-in “Find Duplicates” function and removed after manual verification. Two reviewers independently exported the deduplicated library to screen titles, abstracts, and full texts.

**PubMed search strategy.**

- #1:(“Prediabetic State”[Mesh]) OR ((((Prediabetes[Title/Abstract]) OR (Pre-diabetes[Title/Abstract])) OR (Impaired Glucose Tolerance[Title/Abstract])) OR (Impaired Fasting Glucose[Title/Abstract]))
- #2: (“Behavioral Sciences”[Mesh]) OR (behavior*[Title/Abstract])
- #3: (“Digital Technology”[Mesh]) OR ((((((((Digital Therapeutics[Title/Abstract]) OR (mHealth[Title/Abstract])) OR (eHealth[Title/Abstract])) OR (Telehealth[Title/Abstract])) OR (mobile[Title/Abstract])) OR (internet[Title/Abstract])) OR (digital[Title/Abstract])) OR (online[Title/Abstract]))
- #4:(Type 2 Diabetes[Title/Abstract])
- #5: #1 AND #2 AND #3 NOT #4

### Inclusion and Exclusion Criteria

Studies were included if they met the following criteria: (1) the study population consisted of individuals aged ≥18 years diagnosed with prediabetes based on the diagnostic criteria established by the American Diabetes Association [1]; (2) the intervention involved digital therapeutics grounded in behavioral science and specifically targeted individuals with prediabetes; and (3) the study used any research design, including cross-sectional studies, cohort studies, randomized controlled trials, quasi-experimental studies, or pre-post designs.

The following exclusion criteria were applied: (1) studies involving multiple populations that did not report outcomes for the prediabetes subgroup separately, (2) studies for which the full text was unavailable, and (3) studies not published in Chinese or English.

### Study Selection and Data Extraction

This study’s selection process consisted of an initial screening and a full-text review. First, 2 reviewers (ZP and NZ) independently screened the titles and abstracts based on the predefined inclusion and exclusion criteria. Studies that did not meet the criteria were excluded. Full texts were retrieved and reviewed in detail using the same criteria to determine final inclusion for eligible records. Any disagreements during the screening process were resolved through discussion; if consensus could not be reached, a third and fourth reviewer (QY and XL) were consulted to resolve discrepancies.

Data were extracted from the included studies using a standardized approach. Extracted information included authors, year of publication, country, study design, study population, sample size, intervention duration, theoretical framework, primary forms and components of digital therapeutics, and key outcome measures.

To standardize the identification and reporting of BCTs, we used the behavior change technique taxonomy version 1 (BCTTv1; 93 techniques) [21]. All authors independently identified and mapped intervention elements to BCTTv1 using the official definitions, then resolved discrepancies through group discussion to reach consensus.

## Results

### Selection and Inclusion of Studies

**Figure 1:**
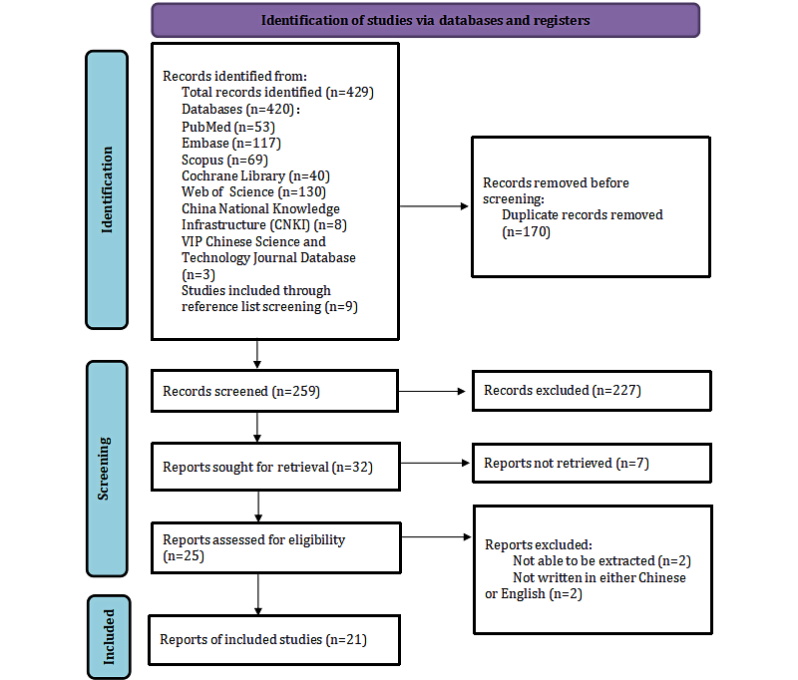
PRISMA flow diagram of study selection. PRISMA: Preferred Reporting Items for Systematic Reviews and Meta-Analyses.

Figure 1 illustrates the PRISMA (Preferred Reporting Items for Systematic Reviews and Meta-Analyses) flow diagram. A total of 429 records were identified through database searching and manual screening. After removing 170 duplicates, 259 unique records remained. Title and abstract screening excluded 227 records, leaving 32 for full-text review. Of these, 11 were excluded: 7 due to full-text unavailability, 2 for not reporting results specific to individuals with prediabetes, and 2 for being published in languages other than Chinese or English. A total of 21 studies were included in the final analysis.

### Characteristics of Included Studies

All 21 included studies spanned from 2013 to 2024; a total of 16 of 21 (76%) studies were published between 2020 and 2024, indicating a growing interest in this topic. All papers were published in English. Two studies were conducted in each of the following locations: China, the United Kingdom, Singapore, and India. One study originated from New Zealand, South Korea, Canada, and Saudi Arabia. One study was a collaborative effort between the United Kingdom and India. The remaining studies (n=8) were conducted in the United States.

In addition to randomized controlled trials (n=17), the included studies comprised 2 quasi-experimental studies, 1 retrospective cohort study, and 1 observational study. The duration of interventions ranged from 28 days to 4 years, with the majority lasting between 3 and 12 months.

An overview of the basic characteristics of the included studies is presented in Table 1.

**Table 1:** Basic information of included studies (n=21).

Study	Year and country	Study type	Population	Sample size（T/C^a^)	Intervention duration (months)	Theoretical model	Digital therapeutic format
Ramachandran et al [22]	2013; India	RCT^b^	Males aged 30-55 years with impaired glucose tolerance	271/266	24	Transtheoretical model (TTM)	Communication platforms
Block et al [23]	2015; United States	RCT	Adults with prediabetes	163/176	6	A-E^c^	Mobile apps and health coach guidance
Fischer et al [24]	2016; United States	RCT	Adults with prediabetes	82/81	12	NS^d^	Communication platforms and health coach guidance
Griauzde et al [10]	2019; United States	RCT	Adults with prediabetes	（24/22）/23^e^	3	Self-determination theory (SDT)	Mobile apps, app-connected devices
Kim et al [25]	2019; United States	Quasi-experimental	Individuals with prediabetes and type 2 diabetes	50	6	Information-Motivation-Behavioral Skills (IMB) self-care model	Mobile apps, app-connected devices, and health coach guidance
McLeod et al [26]	2020; New Zealand	RCT	Individuals with prediabetes and type 2 diabetes	110/115	12	Cognitive behavioral theory (CBT)	Web-based platforms and health coach guidance
Toro-Ramos et al [27]	2020; United States	RCT	Adults with prediabetes	101/99	12	NS	Mobile apps and health coach guidance
Staite et al [28]	2020; United Kingdom	RCT	Individuals aged 18-65 years with prediabetes	98/102	12	Theory of planned behavior (TPB)	Mobile apps, communication platforms, and app-connected devices
Nanditha et al [29]	2020; United Kingdom and India	RCT	Adults with prediabetes	1031/1031	24	Transtheoretical model (TTM)	Communication platforms
Chen et al [30]	2020; China	RCT	Adults with prediabetes	57/43	3	NS	Mobile apps and health coach guidance
Khunti et al [31]	2021; United Kingdom	RCT	Adults with prediabetes	(450/456)/460^e^	48	Social cognitive theory (SCT)	Communication platforms, app-connected devices, and health coach guidance
Muralidharan et al [32]	2021; India	RCT	Individuals aged 20-65 years with prediabetes	271/290	3	NS	Mobile apps, communication platforms, and health coach guidance
Al-Hamdan et al [33]	2021; Saudi Arabia	RCT	Women aged 18-60 years with prediabetes	（40/43）/37^e^	6	NS	Mobile apps, communication platforms, and app-connected devices
Lee et al [34]	2021; South Korea	RCT	Adults with prediabetes	207/208	6	NS	Web-based platforms and app-connected devices
Katula et al [11]	2022; United States	RCT	Adults with prediabetes	299/300	12	NS	Web-based platforms, app-connected devices, and health coach guidance
Batten et al [35]	2022; Canada	Cohort study	Adults with prediabetes	1095	12	NS	Mobile apps, communication platforms, app-connected devices, and health coach guidance
Epstein et al [36]	2022; United States	RCT	Adults with prediabetes	31/33	6	Delay discounting theory	Mobile apps
Lim et al [37]	2022; Singapore	RCT	Adults aged 21-75 years with prediabetes and BMI ≥23	72/76	6	NS	Mobile apps and health coach guidance
Zahedani et al [38]	2023; United States	Quasi-experimental	Individuals with normal glucose, prediabetes, and type 2 diabetes	(746/206/94)/N/A^e,f^	28 days	NS	Mobile apps and app-connected devices
Chung et al [39]	2023; China	RCT	Adults with prediabetes	（41/42）/38^e^	3	NS	Mobile apps, communication platforms, and health coach guidance
Han et al [9]	2024; Singapore	Observational study	Adults with prediabetes and BMI ≥23	148	6	Obesity-Related Behavioral Intervention Trials (ORBIT) model	Mobile apps and health coach guidance

^a^T: treatment group; C: control group.

^b^RCT: randomized controlled trial.

^c^A: learning theory and habit formation models; B: cue- and trigger-centered models; C: social cognitive theory; D: theory of planned behavior; E: delay discounting theory.

^d^NS: not specified.

^e^Values in brackets are for intervention arms, and values outside the brackets are for the control group. For example, “(24/22)/23” indicates three groups: n=24 and n=22 in the two intervention arms, and n=23 in the control group.

^f^N/A: not applicable.

### Theoretical Applications of Behavioral Science in Digital Therapeutics for Individuals With Prediabetes

#### Overview

A review of the included studies reveals that several behavioral theories were applied in designing digital therapeutics targeting individuals with prediabetes. These included SCT [23,31], theory of planned behavior [23,28], transtheoretical model (TTM) [22,29], delay discounting theory [36], Obesity-Related Behavioral Intervention Trials (ORBIT) model [9], cognitive behavioral theory (CBT) [26], self-determination theory (SDT) [10], and Information-Motivation-Behavioral Skills (IMB) self-care model [25]. The TTM was the most frequently used as a single theoretical framework. Notably, 1 study incorporated 5 distinct theoretical models [23].

Additionally, 11 studies used theory-informed BCTs in their intervention design, yet did not explicitly specify the underlying theoretical framework [11,24,27,30,32-35,37-39]. The frequency of theory application is summarized in Table 2.

**Table 2:** Theoretical models applied in the included studies.

Theoretical model	Number of studies	References
Social cognitive theory	2	[23,31]
Theory of planned behavior	2	[23,28]
Transtheoretical model (TTM)	2	[22,29]
Delay discounting theory	1	[36]
Self-determination theory	1	[10]
Information-Motivation-Behavioral Skills (IMB) model	1	[25]
Obesity-Related Behavioral Intervention Trials (ORBIT) Model	1	[9]
Cognitive behavioral theory	1	[26]
Learning theory and habit formation	1	[23]
Cue- and trigger-centered models	1	[23]
Positive psychology	1	[23]

To better understand how behavioral science informs digital interventions, it is essential to examine the theoretical models that underpin these interventions and the specific techniques used to operationalize them. While behavioral theories—such as TTM, SCT, and the theory of planned behavior—provide valuable conceptual frameworks for explaining how and why behavior change occurs, they often offer limited guidance on the concrete and replicable components required for intervention design and implementation.

To address this gap between theory and practice, Michie et al developed the BCTTv1 in 2013 [21]. This consensus-based taxonomy classifies 93 distinct BCTs into 16 groups, each with standardized definitions and coding. BCTs are the “active ingredients” of behavioral interventions, observable, replicable, and irreducible strategies intended to modify behavior by targeting specific psychological mechanisms. The BCTTv1 provides a systematic and transparent framework for researchers, practitioners, and intervention designers to describe, implement, and evaluate behavioral interventions, thereby improving reproducibility, comparability, and scientific rigor.

Analysis of the 21 included studies revealed that digital therapeutic interventions commonly incorporated multiple BCTs during their design.

Among the 93 BCTs identified in the BCTTv1 [21], 36 were applied across the included studies. The 5 most frequently used techniques were self-monitoring of behavior, instruction on how to perform the behavior, goal setting (behavior), information about health consequences, and social support (unspecified). Detailed findings are presented in Table 3.

Notably, all studies combined at least 5 BCTs when designing their interventions, highlighting the complexity and multifaceted nature of behavior change strategies in digital therapeutics.

The following section provides examples of how behavioral theories are applied in practice. It shows how specific theoretical constructs are translated into digital components and BCTs. These cases help demonstrate the real-world use of theory-based interventions for individuals with prediabetes.

**Table 3:** BCTs^a^ used in digital therapeutic interventions.

BCT	BCT code	Frequency	References
Self-monitoring of behavior	2.3	19	[9-11,23-28,30-39]
Instructions on how to perform the behavior	4.1	16	[9,11,22,24-30,32,33,36-39]
Goal setting (behavior)	1.1	15	[9,26-39]
Information about health consequences	5.1	15	[10,22,23,25,27-33,35,37-39]
Social support (unspecified)	3.1	11	[9,11,23,25-28,31,35,37,39]
Prompts or cues	7.1	10	[9,10,22-24,29-31,36,39]
Credible source	9.1	9	[25,27-30,32,36-38]
Feedback on behavior	2.2	8	[9,23,27,28,30,32,38,39]
Social support (practical)	3.2	7	[24,27,32-34,37,39]
Action planning	1.4	6	[11,22,23,29,31,35]
Demonstration of the behavior	6.1	6	[9,24,26,27,36,39]
Adding objects to the environment	12.5	6	[30,33,36-39]
Problem-solving	1.2	5	[24,25,31,32,35]
Goal setting (outcome)	1.3	5	[28,36-39]
Feedback on outcomes of behavior	2.7	5	[27,28,37-39]
Social reward	10.4	5	[25,27,35,36,39]
Self-monitoring of outcome	2.4	4	[10,24,35,37]
Information about social and environmental consequences	5.3	4	[25,29,32,35]
Verbal persuasion about capability	15.1	4	[25,27,36,37]
Review behavior goals	1.5	3	[25,31,39]
Social support (emotional)	3.3	3	[11,24,34]
Information about emotional consequences	5.6	2	[22,31]
Social comparison	6.2	2	[23,31]
Behavioral practice or rehearsal	8.1	2	[30,33]
Material incentive (behavior)	10.1	2	[33,39]
Material reward (behavior)	10.2	2	[23,39]
Restructuring the social environment	12.1	2	[28,29]
Framing or reframing	13.2	2	[10,37]
Review outcome goals	1.7	1	[34]
Information about antecedents	4.2	1	[29]
Behavioral practice or rehearsal	8.2	1	[31]
Graded tasks	8.7	1	[36]
Nonspecific reward	10.3	1	[37]
Restructuring the physical environment	12.2	1	[29]
Avoiding or reducing exposure to cues for the behavior	12.3	1	[22]
Focus on past success	15.3	1	[25]

^a^BCT: behavior change technique.

#### TTM-Guided Stage-Based Messaging

The TTM was applied in 2 studies to guide diabetes prevention interventions. In Ramachandran et al [22], the intervention was structured around TTM’s 5 stages—precontemplation, contemplation, preparation, action, and maintenance [40]. Stage-specific SMS text messages were sent accordingly—for example, messages about the benefits of exercise for those in the precontemplation stage and actionable instructions such as “take a brisk 30-minute walk daily” for those in the action stage. Messages were adjusted every 6 months based on participants’ stages. The intervention group showed a 36% reduction in diabetes risk and improved dietary adherence, supporting the effectiveness of stage-matched interventions.

Nanditha et al [29] used a similar approach, delivering 75-80 messages per stage and incorporating decisional balance through questionnaires. However, after 2 years, no significant difference in diabetes incidence was observed. This may be due to a lower message frequency in this study (2-3 times per week, approximately 8-12 per month) compared with the higher delivery intensity in the Indian trial. Additionally, the Indian trial used HbA_1c_ rather than the oral glucose tolerance test, which may be less sensitive to short-term glycemic changes.

Together, these studies demonstrate the feasibility of TTM-based interventions but suggest that effectiveness may depend on factors such as intensity, evaluation methods, and cultural relevance.

#### SDT in Digital Health

Griauzde et al [10] applied SDT to develop a mobile health app that promotes healthy behaviors in individuals with prediabetes by enhancing intrinsic motivation. Participants were encouraged to log daily habits (eg, sleep, activity, and diet) and reflect on how these aligned with their core values. Personalized feedback and predictive tools supported this process, strengthening the link between behavior and internal values and fostering autonomous motivation.

Although quantitative data showed no significant change in motivation, likely due to already high baseline levels, qualitative interviews revealed increased self-reflection and greater awareness of how behavior relates to one’s environment. The results suggest that future interventions should focus on individuals with lower baseline motivation and include interpersonal support to strengthen SDT’s “relatedness” component, thereby better sustaining behavior change.

#### IMB Model for Diabetes Self-Management

The IMB model, developed by the Fisher brothers [41], emphasizes that effective behavior change requires 3 elements: accurate information, adequate motivation, and practical behavioral skills. Kim et al [25] applied the IMB model, along with persuasive technology, to design a diabetes management intervention.

Information was delivered via a mobile platform offering Korean-language diabetes education and dietary guidance. Motivation was supported through personalized interviews by community health workers and real-time feedback. Behavioral skills were developed through the use of home monitoring devices and stepwise goal setting.

Persuasive techniques—such as tailored prompts and social support—were integrated throughout. For example, abnormal readings triggered human follow-up. After 6 months, 56% of participants achieved normal HbA_1c_ levels, demonstrating the IMB model’s effectiveness in chronic disease management.

#### ORBIT Model in Intervention Design

Han et al [9] used the ORBIT model [42] to design a mobile health intervention. This study applied the full 4-phase ORBIT framework.

In phase I (define), the team identified poor diet quality and self-management barriers in people with prediabetes. Phase II (refine) tested the feasibility of core app functions. Phase III (optimize) involved iterative improvements, including the addition of real-time nutrition advice and social support. In phase IV, a randomized trial demonstrated a significant improvement in dietary quality, with a 6.2-point increase in Alternative Healthy Eating Index–2010 scores in the intervention group (*P*<.01).

This phased approach ensured scientific rigor and facilitated the successful translation of theory into practice.

#### CBT-Based Behavior Change Strategies

CBT [43] served as the foundation of the BetaMe or Melon digital health program, evaluated by McLeod et al [26], which aimed to improve diabetes self-management through structured modules. CBT principles were applied in health coaching (via regular video or audio sessions to reframe negative thinking), goal tracking (using behavioral experiments to monitor diet and activity), and a peer support forum (to promote adaptive coping).

The intervention targeted unhelpful beliefs (eg, “change is impossible”) and reinforced behavior to boost self-efficacy. Although HbA_1c_ improvements were not statistically significant, the intervention group demonstrated gains in secondary outcomes, including self-management confidence. These results support the role of CBT in promoting behavior change and highlight the need for optimized dosage and personalization.

#### Multitheory Integration: Alive-PD Example

Block et al [23] developed the Alive-PD (a fully automated diabetes prevention behavioral intervention program from Turnaround Health, a division of NutritionQuest) by integrating multiple behavioral theories, showcasing an innovative and synergistic approach. Based on SCT [44], the program used digital goal-tracking tools and web-based social support to enhance self-efficacy and reshape environmental influences. The theory of planned behavior [45] guided a personalized goal-setting algorithm by translating attitudes and social norms into actionable steps.

A point-based reward system rooted in behavioral economics [46] provided immediate positive feedback to boost intrinsic motivation. Learning theory supported incremental goal setting with automated reminders as habit cues [47], while positive psychology [48] enhanced achievement and identity through visual progress tracking.

This multi-theory design surpassed the limits of single-model approaches, allowing cognitive, emotional, and environmental pathways to shape behavior through automated, personalized delivery. The results showed that 84% of participants remained highly engaged, and 35% achieved a weight loss of 5% or more, highlighting the benefits of integrated theory in promoting adherence and effective behavior change.

### Forms and Components of Behavioral Science Applications in Digital Therapeutics for Individuals With Prediabetes

#### Overview

The intervention formats could be categorized into single-format and multi-format approaches. Three studies used a single-format intervention, primarily relying on mobile apps or communication software. In contrast, 18 studies used a combination of digital therapeutic formats. See Table 4 for details.

**Table 4:** Intervention formats based on digital therapeutics^a^.

Combination type	Studies, n (%)
a	1 (4.8)
b	2 (9.6)
a+d	2 (9.6)
a+e	5 (23.6)
b+e	1 (4.8)
c+d	1 (4.8)
c+e	1 (4.8)
a+b+d	2 (9.6)
a+b+e	2 (9.6)
b+d+e	1 (4.8)
a+b+d+e	1 (4.8)
c+d+e	1 (4.8)
ade	1 (4.8)

^a^a: mobile app; b: communication software; c: web-based platform; d: app-connected devices; and e: health coach guidance.

Current digital interventions are predominantly mobile-based, with smartphone apps serving as the primary platform. These apps commonly integrate features such as data synchronization and remote health coaching. Communication software and hardware devices serve as supporting components. Communication tools are used for reminders, education, and social support, while hardware devices facilitate data collection.

Common app-connected devices include glucometers, pedometers, wireless scales, and Bluetooth wristbands. As shown in Table 4, digital therapeutic interventions exhibit considerable diversity in format, with most studies adopting a combination of 2 or more delivery modes. The most frequently used configuration involves integrating mobile apps with health coach guidance.

The content of digital therapeutic interventions can be summarized into 6 core modules.

#### Goal Setting

Goal setting reduces psychological barriers to behavior change by breaking long-term objectives into actionable short-term tasks. Various strategies were used across studies. Some used personalized micro-goals based on questionnaire analysis, with systems automatically generating weekly targets in physical activity, diet, and psychosocial areas according to individual profiles [23]. Others combined web-based skills training with motivational interviewing to provide participants with diabetes self-management education and individualized goal counseling, supporting healthy lifestyle behaviors [25]. Stage-matched SMS text messaging interventions grounded in the TTM delivered periodic messages offering prompts, advice, and reinforcement strategies related to goal setting, physical activity, diet, and overall lifestyle change [29,31]. Web- and app-based goal tracking was also common: for example, Lee et al [34] used a website to tailor weight loss or maintenance goals and daily caloric intake based on participants’ body weight status; Epstein et al [36] used the “MyFitnessPal” (MyFitnessPal, Inc) app, requiring participants to set calorie goals and adhere to a traffic light-based dietary system (<2 servings of red-light foods and >5 servings of green-light foods daily), with exercise goals of at least 150 minutes of moderate or 75 minutes of vigorous activity per week. Lim et al [37] implemented a step-counting app that automatically increased daily step targets, progressing from 3000 steps per day in week 1 to 10,000 steps by week 3.

#### Self-Monitoring

Self-monitoring enhances individuals’ awareness of their behaviors through the collection and analysis of multidimensional health data. Common strategies included app-based tracking of sleep, diet, and physical activity [10,27,28,30,35-39], as well as the use of home-based monitoring devices to record weight, blood glucose, blood pressure, dietary intake, and exercise [11,25,31,34]. Several interventions provided personalized recommendations based on data from wearable devices [37-39]. For example, Lim et al [37] used an app with automated response features that assessed dietary choices and generated culturally tailored food substitution lists. Two studies [38,39] used AI-driven algorithms to deliver personalized suggestions on diet and physical activity, including reducing calorie intake, increasing dietary fiber and protein, and promoting physical activity. Notably, 1 study [39] incorporated principles of traditional Chinese medicine, using body constitution analysis and meridian energy profiling to tailor lifestyle recommendations further.

#### Real-Time Feedback and Behavioral Reinforcement

Grounded in behavioral economics principles, real-time feedback and reward systems were implemented to enhance motivation and reinforce behavior. Strategies included point-based rewards and team competitions, where participants earned points by completing weekly goals that could be redeemed for incentives or used to support team challenges [23]. Automated feedback mechanisms were integrated into behavior change programs, such as the “Noom program” (Noom, Inc), which delivered daily tasks (eg, food selection) and evaluated responses in real-time to provide immediate feedback [27]. Some studies also used in-app reward points (eg, “virtual gold”), allowing participants to earn rewards by completing questionnaires, quizzes, or personal goals, which could then be exchanged for tangible incentives [39]. Feedback was further delivered through SMS text messaging and app-based notifications [25,35,37], as well as real-time data syncing from wearable devices [11,28,31]. For example, Kim et al [25] provided participants with Bluetooth-enabled home monitoring devices (eg, glucometers, blood pressure monitors, pedometers, and scales), which automatically transmitted data to a smartphone app. Similarly, in studies by Katula et al [11] and Batten et al [35], wearable trackers and smart scales synced automatically with the intervention platform, facilitating continuous behavioral feedback.

#### Social Support and Group Interaction

Social networks—both digital and offline—were leveraged to enhance adherence to interventions and behavioral engagement. Strategies included web-based team-based competitions to foster peer accountability [23], as well as mobile app-based coach-participant interactions through mobile platforms. For instance, Toro-Ramos et al [27] enabled participants to access coach and group messages via mobile devices and communicate directly with coaches as needed. Similarly, in studies by Katula et al [11] and Batten et al [35], participants engaged in web-based group discussions and in-app community interactions to ask questions, responded to peer feedback, and shared personal progress. Additional approaches included follow-up phone calls to maintain participant engagement [11,31,32] and the provision of gym memberships to promote physical activity through environmental and social reinforcement [33]. In that study, Al-Hamdan et al [33] offered free gym access to all participants in the intervention group. Remote dietary counseling was also provided in several studies [9,30,37], where participants recorded data on their diet, glucose levels, sleep patterns, weight, and physical activity using an app, and nutritionists delivered tailored feedback. In the study by Lim et al [37], nutritionists reviewed submitted data regularly. They used motivational interviewing techniques to provide real-time feedback and help participants overcome behavioral barriers to sustainable change.

#### Reminders and Prompts

Multichannel reminder systems were used to counteract memory decay and promote adherence. Scheduled text message delivery was one of the most common strategies, with messages sent at fixed times according to participant preferences [10,22-26,28,29,31]. For example, Ramachandran et al [22] and Nanditha et al [29] structured messages around the 5 stages of the TTM (precontemplation, contemplation, preparation, action, and maintenance), delivering 60-80 stage-matched messages in cycles. These messages were tailored based on participants’ physical activity and dietary data. In the study by Nanditha et al [29], bilingual texts were used to accommodate diverse racial and cultural backgrounds. App-based motivational messaging was also used to support behavior change. In a study by Staite et al [28], motivational interviewing principles were integrated into a smartphone app that sent automated messages to facilitate health intention formation, encourage self-monitoring, and enhance social support. Additionally, graphical progress dashboards were used to provide visually enriched feedback and reinforce engagement throughout the intervention period [32].

#### Health Education

A structured knowledge delivery chain—from basic awareness to applied practice—was embedded into many interventions. Common formats include general health information messaging covering healthy lifestyles, the benefits of physical activity and nutrition, and diabetes prevention strategies [22-25,28-30,33,35]. One study incorporated the Diabetes Prevention Program–certified educational content [27]. Video-based courses were also used in 2 studies [32,37], both of which delivered 3-month curricula. In the study by Muralidharan et al [32], the content was presented in a reality television format, accompanied by weekly feedback calls from health coaches. In contrast, Lim et al [37] provided videos on weight management, diabetes prevention, healthy meal planning, and food selection. Web-based courses and toolkits were provided in another study [34], featuring an 18-session, 24-week program that guided participants through lifestyle modification, diet, physical activity, and self-monitoring, with supplementary materials and offline coaching. Additionally, Katula et al [11] implemented a year-long stepped curriculum, including 16 weeks of intensive weight-loss content followed by 36 weeks of maintenance-focused education.

### Evaluation Metrics and Outcomes of Behavioral Science Applications in Digital Therapeutics for Individuals With Prediabetes

The primary outcome measures reported in the included studies fell into 5 main categories, as detailed in Table 5.

**Table 5:** Outcome measures reported in the included studies.

Outcome measures		References
**Core glycemic control metrics**		
	Diabetes incidence	[22,29]
	Change in fasting plasma glucose	[23,29,30,32,34,37,39]
	Change in hemoglobin A_1c_ (HbA_1c_)	[11,23-28,30,33,34,36,37,39]
	Time in hyperglycemia	[30,38]
**Metabolic and body composition**		
	Body weight	[11,23-31,33-38]
	BMI	[22,23,26,29-31,34,37,39]
	Waist circumference	[22,23,26,28,29,31,32,34]
	Lipid profile	[11,22,23,25,28-30,32-34,37]
**Cardiovascular and physiological**		
	Blood pressure	[11,22,24-26,28,29,31,32,34,37]
	Physical activity levels	[22,28-31,35-39]
**Behavioral and cognitive**		
	Dietary quality	[9,22,29,30,33,37-39]
	Delay discounting	[36]
	Intrinsic motivation	[10]
	Self-efficacy	[25]
	Diabetes knowledge	[25]
Comprehensive health outcomes: quality of life		[26,29]

Most studies reported at least one primary or secondary outcome measure that reached statistical significance. The most frequently reported outcomes included body weight, HbA_1c_, fasting plasma glucose, lipid profiles, blood pressure, physical activity levels, waist circumference, and BMI. However, the findings across studies were heterogeneous.

Among the 16 studies that assessed body weight [11,23-31,33-38], only 50% reported statistically significant differences between the intervention and control groups. Of the 13 studies examining changes in HbA_1c_, fewer than half [11,23,25,27,37] found significant improvements. Regarding fasting plasma glucose, only 2 of the 7 relevant studies [23,37] observed meaningful reductions.

For lipid profiles, 5 of 11 studies reported significant changes, with 2 studies [22,34] identifying improvements solely in high-density-lipoprotein cholesterol levels. Among the 11 studies addressing blood pressure, 3 reported improvements, of which 2 [24,32] found significance only in systolic blood pressure.

Physical activity improvements were observed in 2 of 10 studies [35,38]. For waist circumference, 4 of 8 studies [23,26,32,34] reported statistically significant differences. Among 9 studies assessing BMI, 4 [23,26,34,37] found significant reductions. Notably, 1 study [26] reported a significant effect at the 4-month follow-up, which was no longer observed at 12 months.

To enhance the clarity and interpretability of the findings, Multimedia Appendix 3 [9,10,22,23,25,26,28,29,31]: integration of theories, techniques, and outcomes presents an integrated table that systematically maps the relationships among behavioral theories, applied BCTs, digital intervention components, and health outcomes, further elucidating the connection between theoretical foundations and practical implementation.

### Limitations of Included Studies

Several important considerations emerged when interpreting the included studies, including variability in theories and frameworks, sample characteristics, data collection methods, and intervention duration. These factors may influence the strength and generalizability of the evidence and should be acknowledged when evaluating the findings.

### Unclear Application of Theoretical Frameworks

Most included studies incorporated BCTs, offering valuable guidance for designing actionable strategies in digital interventions. A total of 11 studies applied BCTs commonly linked to behavioral science [11,24,27,30,32-35,37-39], yet did not identify the underlying theoretical frameworks. This may suggest that researchers, as interdisciplinary practitioners, had a limited depth of understanding in behavioral science, which resulted in insufficiently precise or standardized articulation of the theoretical underpinnings. Notably, even when theories were not explicitly named, the BCTs used—such as self-monitoring, goal setting, feedback, and social support—can be traced to established frameworks such as SCT, the theory of planned behavior, and SDT.

### Limited Application of Theoretical Frameworks

Among the studies that did report guiding theories, most relied on a single theoretical model. While such models (eg, the theory of planned behavior) offer functional constructs for understanding behavioral intention, they may fall short in capturing the complexity of behavior change in real-world contexts [49]. Additionally, commonly used models such as the TTM may define behavioral stages too broadly, limiting the ability to track nuanced changes over time [50]. These observations suggest that future digital interventions could benefit from more explicit theoretical articulation and potentially from integrating multiple complementary models to enhance conceptual rigor and intervention effectiveness.

### Short Durations and Small Samples

Most interventions had short durations ranging from 1 to 6 months [9,10,23,25,30,32-34,36-39], with limited follow-up periods, thereby restricting the assessment of long-term behavioral and clinical outcomes. This may reflect the practical and resource-related challenges of sustaining user engagement in long-term digital interventions, especially after discharge, when continuous monitoring is required, and the absence of personalized feedback or human interaction further hinders participation [51-53]. Additionally, many studies had relatively small sample sizes (n<100) and high attrition rates [10,33,37,39], which may compromise statistical power and increase the risk of bias in effect estimation [54].

### Limitations in Data Accuracy and Population Representativeness

The accuracy of self-reported data and sensor-based tracking was a common concern, with underreporting and technical errors noted in several studies [10,28,38]. For instance, calorie intake in dietary self-reports is commonly underestimated by 20%-30% [55]. Additionally, most interventions targeted younger populations, limiting the generalizability to older adults and underserved groups [10,11,25,33,35,39].

## Discussion

### Principal Findings

This review, grounded in behavioral science, systematically examined 21 clinical studies of digital therapeutics for individuals with prediabetes, focusing on theoretical frameworks, technological implementation, intervention strategies, and clinical outcomes. The findings indicate that integration between behavioral science and digital therapeutics is emerging, especially in structured module design and the application of BCTs. However, the optimization and scalability of current interventions remain limited due to insufficient integration between behavioral theories and digital functionalities, a lack of theoretical model diversification, and limited applicability across varied populations and real-world contexts.

Our results reaffirm the critical role of theory in guiding behavior change, which aligns with previous research [56,57]. Widely adopted models, such as TTM, SCT, and the theory of planned behavior, are consistent with prior bibliometric analyses of digital health behavior change interventions [58]. However, despite the availability of these well-established models, most studies relied on single-theory frameworks, with limited attempts to integrate multiple models. Among the 21 studies included, only 1 incorporated multiple theoretical frameworks. This multi-theory framework illustrates how combining theoretical constructs with digital components can yield more robust, personalized, and engaging interventions. A recent systematic review also found a positive association between using multiple theories and improved intervention outcomes [59]. While the integration of multiple theories may enhance intervention effectiveness, it is important to balance the complexity and usability of interventions, as excessive intervention components may reduce user engagement [7,60].

The results of this review indicate that self-monitoring, goal setting, feedback, and social support are the most frequently used and effective BCTs. Digital platforms integrating mobile apps with health coaching or peer-support networks generally demonstrate better outcomes. Therefore, future digital intervention designs should incorporate these validated elements.

Digital therapeutics for prediabetes are delivered through various platforms, including mobile apps, wearable sensors, SMS-based text messaging systems, web portals, and professional coaching. Effective interventions often combine these technologies. For example, apps connected to fitness trackers provide real-time activity feedback; SMS text messaging or in-app messaging delivers health education and behavioral prompts; web-based platforms support peer discussion; and videoconferencing or chat functions enable tailored coaching. Studies show multicomponent programs combining behavior tracking, health education, and professional guidance yield better clinical outcomes [61]. This suggests that technology must be purposefully aligned with behavioral strategies.

However, the results remain heterogeneous despite most studies reporting statistically significant improvements in at least 1 primary or secondary outcome. Commonly reported outcomes include body weight, HbA_1c_, fasting plasma glucose, lipid profiles, BMI, blood pressure, physical activity, and waist circumference. Among these, body weight and HbA_1c_ were the most frequently measured indicators. Still, inconsistencies exist: for example, 2 studies evaluating ≥3% weight loss produced conflicting conclusions [28,62]. Variability in study populations, intervention modalities, measurement tools, and follow-up periods further complicates direct comparisons and limits generalizability.

### Limitations and Future Directions

Building on the discussion of theoretical and technological integration, it is also important to acknowledge the limitations of the current evidence base and identify opportunities for future research. Although a systematic literature search was conducted, the restriction to Chinese and English publications may have omitted relevant studies published in other languages. Given the limited number of relevant studies, a systematic review or meta-analysis was not feasible. We conducted a scoping review to map existing evidence and descriptively summarize key limitations. Literature screening and data extraction were performed collaboratively by multiple reviewers to enhance the reliability of the process. Future systematic reviews or meta-analyses may consider incorporating formal appraisal tools to improve methodological rigor as evidence grows. At present, substantial heterogeneity in study design, theoretical frameworks, and outcome measures limits the comparability and external validity of the findings.

Furthermore, many existing studies on digital therapeutics for prediabetes have been conducted in high-income Western countries, such as the United States and parts of Europe, with fewer studies emerging from high-income Asian regions. This distribution likely reflects broader global trends in digital health research, including greater investment in digital infrastructure and higher levels of digital literacy in these settings. However, the geographic concentration of current evidence limits the generalizability of findings to low- and middle-income countries (LMICs), where digital health interventions may encounter different contextual barriers—such as limited internet access, lower smartphone penetration, and diverse sociocultural attitudes toward technology use. These factors may influence digital therapeutics’ implementation, scalability, and effectiveness in LMIC contexts. Future research should prioritize the inclusion of more diverse populations and settings. Specifically, studies should explore the cultural adaptability, linguistic accessibility, and feasibility of digital health interventions in LMICs. It is also essential to address systemic barriers—such as digital divides and limited health literacy—that may hinder adoption. Intervention design should be informed by cultural sensitivity and equity principles, including language diversity and contextual relevance considerations.

Moreover, future research should focus on advancing both theoretical and technological innovation. This includes integrating multiple behavioral science theories and applying intelligent technologies to enable personalized intervention pathways, adaptive feedback mechanisms, and sustained user engagement. Under the guidance of theories, further efforts are needed to develop classification frameworks for behavioral constructs and decision-support tools to enhance the rigor of theory selection and the practicality of intervention strategies [63]. Additionally, machine learning techniques could be leveraged to identify and optimize combinations of BCTs tailored to specific populations, thereby improving intervention effectiveness and facilitating broader implementation.

In conclusion, this scoping review indicates that behavioral science theories play a substantive role in designing digital therapeutics for prediabetes. Across various modalities, including smartphone apps, messaging, wearables, web platforms, and human coaching, BCTs such as self-monitoring, goal setting, and social support are consistently applied. Explicitly explaining the selection and implementation of BCTs based on behavioral theories provides clearer justification and support for research design. Future studies should broaden cultural representation and include a wider range of age groups to improve generalizability and scalability.

## Appendix

Multimedia Appendix 1 PRISMA-ScR checklist.

Multimedia Appendix 2 Search terms used to identify studies.

Multimedia Appendix 3 Integration of theories, techniques, and outcomes.

## Abbreviations

BCT: behavior change technique
BCTTv1: behavior change technique taxonomy version 1
CBT: cognitive behavioral theory
HbA_1c_: glycated hemoglobin A_1c_
IDF: International Diabetes Federation
IMB: Information-Motivation-Behavioral Skills
LMIC: low- and middle-income country
ORBIT: Obesity-Related Behavioral Intervention Trials
PRISMA: Preferred Reporting Items for Systematic Reviews and Meta-Analyses
PRISMA-ScR: Preferred Reporting Items for Systematic Reviews and Meta-Analyses extension for Scoping Reviews
SCT: social cognitive theory
SDT: self-determination theory
TTM: transtheoretical model

## Data Availability

Data sharing does not apply to this paper as no datasets were generated or analyzed during this study.
