# Supplementary material for: Application of Behavioral Science in Digital Therapeutics for Individuals With Prediabetes: Scoping Review
Source: J Med Internet Res. 2025 Sep 29;27:e78891. doi: 10.2196/78891 (PMC12519023; doi:10.2196/78891)
Supplement: Multimedia Appendix 2 [file jmir_v27i1e78891_app2.doc]

| Database | Search Terms | Hits |
| --- | --- | --- |
| PubMed | #1 ("Prediabetic State"[Mesh]) OR ((((Prediabetes[Title/Abstract]) OR (Pre-diabetes[Title/Abstract])) OR (Impaired Glucose Tolerance[Title/Abstract])) OR (Impaired Fasting Glucose[Title/Abstract])) #2 ("Behavioral Sciences"[Mesh]) OR (behavior*[Title/Abstract]) #3 ("Digital Technology"[Mesh]) OR ((((((((Digital Therapeutics[Title/Abstract]) OR (mHealth[Title/Abstract])) OR (eHealth[Title/Abstract])) OR (Telehealth[Title/Abstract])) OR (mobile[Title/Abstract])) OR (internet[Title/Abstract])) OR (digital[Title/Abstract])) OR (online[Title/Abstract])) #4 (Type 2 Diabetes[Title/Abstract]) #5 #1 AND #2 AND #3 NOT #4 | 53 |
| Embase | #1 'prediabetic state':ti,ab,kw OR prediabetes:ti,ab,kw OR 'pre diabetes':ti,ab,kw OR 'impaired glucose tolerance':ti,ab,kw OR 'impaired fasting glucose':ti,ab,kw #2 'behavioral sciences':ti,ab,kw OR behavior*:ti,ab,kw #3 'digital technology':ti,ab,kw OR 'digital therapeutics':ti,ab,kw OR mhealth:ti,ab,kw OR ehealth:ti,ab,kw OR telehealth:ti,ab,kw OR mobile:ti,ab,kw OR internet:ti,ab,kw OR digital:ti,ab,kw OR online:ti,ab,kw #4 #1 AND #2 AND #3 | 117 |
| Scopus | #1 ( TITLE-ABS-KEY ( Prediabetic State ) OR TITLE-ABS-KEY ( Prediabetes ) OR TITLE-ABS-KEY ( Pre-diabetes ) OR TITLE-ABS-KEY ( Impaired Glucose Tolerance ) OR TITLE-ABS-KEY ( Impaired Fasting Glucose ) ) #2 ( TITLE-ABS-KEY ( Behavioral Sciences ) OR TITLE-ABS-KEY ( behavior* ) ) #3 ( TITLE-ABS-KEY ( digital technology ) OR TITLE-ABS-KEY ( Digital Therapeutics ) OR TITLE-ABS-KEY ( mHealth ) OR TITLE-ABS-KEY ( eHealth ) OR TITLE-ABS-KEY ( Telehealth ) OR TITLE-ABS-KEY ( mobile ) OR TITLE-ABS-KEY ( internet ) OR TITLE-ABS-KEY ( digital ) OR TITLE-ABS-KEY ( online ) ) #4 TITLE-ABS-KEY ( Type 2 Diabetes ) #5 #1 AND #2 AND #3 NOT #4 | 69 |
| Cochrane Library | #1 (Prediabetes):ti,ab,kw OR (Pre-diabetes):ti,ab,kw OR (Impaired Glucose Tolerance):ti,ab,kw OR (Impaired Fasting Glucose):ti,ab,kw #2 (Behavioral Science):ti,ab,kw OR (behavior*):ti,ab,kw #3 (Digital Therapeutics):ti,ab,kw OR (mHealth):ti,ab,kw OR (eHealth):ti,ab,kw OR (Telehealth):ti,ab,kw OR (mobile):ti,ab,kw OR (internet):ti,ab,kw OR (digital):ti,ab,kw OR (online):ti,ab,kw #4 (Type 2 Diabetes):ti,ab,kw #5 #1 AND #2 AND #3 NOT #4 | 40 |
| Web of Science | #1 TS=(Prediabetic State OR Prediabetes OR Pre-diabetes OR Impaired Glucose Tolerance OR Impaired Fasting Glucose) #2 TS=(Behavioral Sciences OR behavior*) #3 TS=(Digital Technology OR Digital Therapeutics OR mHealth OR eHealth OR Telehealth OR mobile OR internet OR digital OR online) #4 TS=(Type 2 Diabetes) #5 #1 AND #2 AND #3 NOT #4 | 130 |
| CNKI | （篇关摘：糖尿病前期 + 前期糖尿病 + 空腹血糖受损 + 糖耐量减低(精确)）AND（篇关摘：行为科学 + 行为(精确)）AND（篇关摘：数字技术 + 移动健康 + 移动 + 数字健康 + 数字 + 互联网 + 线上(精确)） | 8 |
| VIP | (((((题名或关键词=糖尿病前期 OR 题名或关键词=前期糖尿病) OR 题名或关键词=空腹血糖受损) OR 题名或关键词=糖耐量减低) AND (题名或关键词=行为科学 OR 题名或关键词=行为)) AND ((((((题名或关键词=数字技术 OR 题名或关键词=移动健康) OR 题名或关键词=移动) OR 题名或关键词=数字健康) OR 题名或关键词=数字) OR 题名或关键词=互联网) OR 题名或关键词=线上)) | 3 |
