# Supplementary material for: Application of Behavioral Science in Digital Therapeutics for Individuals With Prediabetes: Scoping Review
Source: J Med Internet Res. 2025 Sep 29;27:e78891. doi: 10.2196/78891 (PMC12519023; doi:10.2196/78891)
Supplement: Multimedia Appendix 3 [file jmir_v27i1e78891_app3.doc]

Multimedia Appendix 3. Integration of Theories, Techniques, and Outcomes.

| Theory | Behavior Constructs | Corresponding BCT | Digital Component Examples | Key Results | Study Exam-ples |
| --- | --- | --- | --- | --- | --- |
| Transtheoretical Model (TTM) | Stages of Behavior Change | Stage-specific feedback, tailored information | Personalized text messages | Improved HbA1c and increased DPP engageme-nt | Ramachandran et al. [22]; Nanditha et al. [29] |
| Self-Determination Theory (SDT) | Intrinsic Motivation | Feedback, self-reflection, self-monitoring | App-based value reflection module | Significant weight loss (≥5%) | Griauzde et al. [10] |
| Information-Motivation-Behavioral Skills (IMB) Model | Information-Motivation-  Behavioral Skills | Educational information, motivation reinforcement, skills training | Educational push messages and interactive online exercises | Reduced fasting glucose and improved lifestyle knowledge | Kim et al. [25] |
| Obesity-Related Behavioral Intervention Trials (ORBIT) Model | Intervention Development and Evaluation Framework | Goal setting, feedback, randomized trials | Stage-based app development and optimization | Improved interventi-on design and sustained behavior change | Han et al. [9] |
| Cognitive Behavioral Theory (CBT) | Cognitive Restructuring | Behavioral experiments, social support, emotional management | In-app cognitive-beh-avioral training modules | Decreased BMI and improved coping skills | McLeod et al. [26] |
| Theory of Planned Behavior (TPB) | Attitude, Subjective Norms, Perceived Behavioral Control | Goal setting, intention formation, planning | Goal-driven digital planning module | Improved intention to exercise and dietary changes | Block et al. [23]; Staite et al. [28] |
| Social Cognitive Theory (SCT) | Self-efficacy, observational learning | Self-monitoring, modeling, reinforcement | Social interaction platform, self-monitori-ng logs | Enhanced physical activity and behavior adherence | Block et al. [23]; Khunti et al. [31] |
| Integration of Multiple Theories | Rewards, Motivation, Social support | Incentives, nudges, goal-setting, feedback | In-app reward system, social interaction, reminder systems | Increased adherence and significant weight reduction | Block et al. [23] |
